# The Journal-Record Incorporated

**Published:** 1904-11

**Authors:** 


### THE JOURNAL-RECORD INCORPORATED.

THE owners of THE ATLANTA JOURNAL-RECORD OF MEDICINE, Miller B. Hutchins, Bernard Wolff and Marian Hillyer Wolff, have filed application for charter for the Atlanta Medical-Journal Company, with a paid-up capital of $3,000, with privilege of increasing it to $10,000.

---

THE new annex of the Tabernacle Infirmary was opened on Monday, October 17.

---

DR. CHAS. E. MURPHEY, who was recently injured in an automobile accident, is recovering.

---

THE Atlanta College of Physicians and Surgeons is to erect new college buildings at a cost of $100,000.

